# Pain is a Limiting Factor in Patients Suitable for Transilluminated Powered Phlebectomy

**DOI:** 10.5041/RMMJ.10377

**Published:** 2019-10-29

**Authors:** Alexander Kantarovsky, Dmitri Vinogradski, Evgenia Mankowitsch, Itamar Ashkenazi

**Affiliations:** Vascular Surgery Department, Hillel Yaffe Medical Center, Hadera, Israel

**Keywords:** Minimally invasive phlebectomy, phlebectomy, transilluminated powered phlebectomy, tumescent anesthesia, varicose veins

## Abstract

**Objectives:**

To analyze, perioperatively and in follow-up, transilluminated powered phlebectomy (TIPP), a surgical technique for the treatment of varicose veins.

**Method:**

Retrospective study in one medical institution of patients undergoing TIPP between July 2015 and December 2017. Data analyzed included demographic data, surgery, and results. Postoperatively, pain was evaluated by a 10-point visual analogue scale. The Venous Clinical Severity Score (VCSS) was assessed 5–8 weeks following surgery.

**Results:**

Sixty-six patients with extensive varicosities who underwent TIPP were included. Postoperative pain scores were higher in patients undergoing bilateral compared to unilateral TIPP (visual analogue score 7 versus 5; P=0.031). Following surgery, the VCSS improved in 81.8% (54/66) of the patients. However, 39.7% (25/63; data missing in 3 patients) reported that they would not be willing to undergo a similar procedure in the future. Pain was the most common reason for dissatisfaction.

**Conclusions:**

Transilluminated powered phlebectomy was associated with considerable pain and discomfort in many patients included in this study. For this reason, it should be reserved for a select group of patients in whom other treatment options are limited; TIPP could be considered in the following cases: patients with a large number of varicosities, reoperations, after extensive thrombophlebitis, obesity, or following bariatric surgery.

## INTRODUCTION

Transilluminated powered phlebectomy (TIPP) is a minimally invasive surgery performed in order to remove varicose veins in the legs. This surgery consists of three components: (1) tumescent anesthesia by which local anesthesia of a large area is achieved by infiltrating the subcutaneous tissue with large volumes of anesthetic solution; (2) transillumination with a light source that enables accurate location of the varicosities, resulting in fewer overlooked diseased veins; and (3) a tissue resector device similar to that used in arthroscopic surgery that removes the varicose veins. The TIPP technique is an additional surgical method for the removal of varicose veins. This technique was successfully used to treat large venous ulcers and was more efficient than the classical phlebectomy.^1^ Compared with other methods, the theoretical advantages of TIPP are: surgery performed under local anesthesia, reduced duration of the procedure, fewer incisions, and the ability to easily identify the varicose veins.

Though the method has been over 20 years in use, the number of articles regarding TIPP is small.^1^–^12^ Most of these show that the surgery can be performed within a day-care setting, the complications are few, and the overall results are satisfactory. Nevertheless, in a randomized study, Chetter et al. compared 29 patients who had TIPP to 33 patients who underwent multiple stab incision phlebectomy.^6^ Their conclusion suggested that TIPP results were worse for pain and quality of life 6 weeks after surgery. This study revealed that recovery from TIPP surgery took longer.

At the Hillel Yaffe Medical Center in Hadera, Israel, we have been performing TIPP since 2015 in patients with abundant varicose veins, and/or previous surgery, and/or a previous event of superficial thrombophlebitis, and especially in obese persons. All the procedures are performed only under tumescent anesthesia.

The objective of this study was to carry out a retrospective review of the results of this procedure performed between July 2015 and December 2017. Pain and patient satisfaction following surgery were investigated.

## METHODS

This was a retrospective study authorized by the local Institutional Review Board. The authors were exempted from receiving informed consent. Included were patients from one medical center who underwent TIPP between July 2015 and December 2017. Patients who did not have a minimum of 4 weeks’ follow-up were excluded.

All the patients included in this study underwent TIPP only. This procedure has been previously described in detail.^13^–^15^ As a policy, patients who presented with varicose veins and concomitant axial (saphenous) vein valvular incompetence underwent staged procedures with main axial vein ablation preceding TIPP. Any adverse events described in this series represent complications directly associated with TIPP and cannot be attributed to an associated procedure.

The patients included in this study received premedication with a sublingual tablet of lorazepam (1 mg) and an oral tablet of clonidine (0.15 mg). Sublingual administration of the benzodiazepine allows quick absorption and rapid onset of its anxiolytic action. Clonidine inhibits tachycardia which may arise secondary to anxiety but also to the tumescent anesthetic solution which includes adrenaline. All underwent surgery using tumescent anesthesia without sedation in order to get their full cooperation during surgery. Since the patients selected for this procedure have a significant quantity of varicose veins distributed to the thighs and legs, this allows changing their position during the procedure as necessary without breaking sterility. All the patients were treated with postoperative compression placed at the end of the procedure. Compression was achieved by placing the following layers: cotton wool, elastic bandage, stockinet, and elastic hosiery. These were removed in the clinic four days following surgery. An elastic stocking was advised as long as the patient felt comfortable using it for symptomatic relief. Following surgery, patients underwent follow-up in the outpatient clinic for at least one year. During this follow-up patients were assessed with standardized clinical assessments based upon patient interview and physical examination. Duplex examination is only indicated if deep vein thrombosis is suspected. Follow-up included patient satisfaction surveys.

Data collected and analyzed included demographic data (age, gender), surgery (indication, time, unilateral/bilateral), and results (complications, residual varicosities). Upon termination of the procedure, operative pain was measured on a 10-point visual analogue scale (0 indicating no pain to 10 indicating worst pain ever). The Venous Clinical Severity Score (VCSS) was assessed 5–8 weeks following surgery.^16^ We used descriptive statistics to analyze the data.

## RESULTS

Sixty-six patients are reported. Forty-seven (71.2%) of the TIPP procedures were performed in females, and 19 (28.8%) were performed in males. Thirty-four underwent unilateral procedures, and 32 underwent bilateral procedures. Comparison of patients undergoing unilateral to those undergoing bilateral TIPP is presented in Table 1.

**Table 1:** Comparison of Unilateral to Bilateral Transilluminated Powered Phlebectomy (TIPP).

Demographics and Surgical Details	Unilateral TIPP (*n*=34)	Bilateral TIPP (*n*=32)	All Patients	*P* Value Unilateral versus Bilateral
Median age (range), y	57 (25–72)	48.5 (26–84)	50.5 (25–84)	0.095

Gender				
Females, *n* (%)	25 (53.2)	22 (46.8)	47 (71.2)	0.889
Males, *n* (%)	9 (47.4)	10 (52.6)	19 (28.8)	

Median length of surgery (range), minutes	61.5 (27–116)	83 (24–142)	72.5 (24–142)	<0.001

Median pain score (range)	5 (0–10)	7 (2–10)	6 (0–10)	0.031

VCSS*				
Before surgery	6 (2–16)	7 (2–13)	6 (2–16)	0.096
After surgery	3 (0–9)	4 (0–17)	3 (0–17)	0.049

* Data on three patients undergoing unilateral TIPP missing.

TIPP, transilluminated powered phlebectomy; VCSS, Venous Clinical Severity score.

Following surgery, VCSS improved in 81.8% (54/66) of the patients. In patients undergoing unilateral TIPP, VCSS after surgery improved in 85.3% (29/34) and worsened in 5.9% (2/34) of the patients. Information concerning VCSS change was missing in 3 patients undergoing unilateral TIPP. In patients undergoing bilateral TIPP, VCSS after surgery improved in 78.1% (25/32), remained the same in 9.4% (3/32), and worsened in 12.5% (4/32) of the patients. Median change in VCSS for unilateral TIPP was similar to median change in VCSS for bilateral TIPP (3.5 versus 3; *P*=0.959).

When asked if they would be willing to undergo a similar procedure in the future, 39.7% (25/63) of the patients responded they would not (data missing in 3 of 66 patients); no difference was noted between patients undergoing unilateral TIPP or bilateral TIPP (38.7% versus 40.6%; *P*=1.000). Pain was reported by 48% (12/25) of those unwilling to undergo a similar procedure in the future; no difference was found between patients undergoing unilateral TIPP and bilateral TIPP (41.7% versus 53.8%; *P*=0.695). Only one patient in this series required additional surgical phlebectomy due to varicose veins missed during the initial TIPP.

## DISCUSSION

A varicose vein is a common condition affecting over one-fifth of the adult population.^17^ In this study we describe our experience with TIPP in selected patients with extensive varicosities. Additionally, most of the patients were obese, had undergone previous venous surgery, or suffered from extensive thrombophlebitis in the past. An important finding of this study was that pain was common and it was a major reason for patient dissatisfaction.

In the patients included in this study, simple phlebectomy and ultrasound-guided foam sclerotherapy do not offer a real alternative to TIPP. In these patients, simple phlebectomy is a very long operation with high risk for wound infection. Veins that need to be removed during simple phlebectomy may be missed since these are not always visible during the procedure. Ultrasound-guided foam sclerotherapy in this group of patients is a long-drawn-out procedure that necessitates several sessions with intervals of weeks between each one. This type of treatment is associated with multiple episodes of local, painful thrombophlebitis and more recurrences.

In our unit, all TIPP procedures were performed under tumescent anesthesia in order to avoid the untoward effects of general anesthesia. Furthermore, tumescent anesthesia facilitates rotating the patients’ extremities safely without breaking sterility. Rotating the patients avoids missing varicosities in the dependent areas. This is crucial in patients with extensive varicosities who are obese. Avoiding general and regional anesthesia allows patients to ambulate soon after the procedure.

Following surgery, these patients were treated similarly to other patients operated for varicose veins, by compression bandages and oral analgesics as needed. Postoperative pain treatment was based upon non-steroidal anti-inflammatory drugs (etodolac 500 mg b.i.d.) and tailored according to currently accepted guidelines.^18^ Nevertheless, our experience shows that this procedure is accompanied with significant pain during and after the operation, even though we used oral sedation and distractive interventions.^19^ Pain was the major reason for dissatisfaction in our patients, many of whom reported that they would not be willing to undergo this procedure again. Before undergoing TIPP, patients should be well informed about the levels of pain that could be experienced during and after this procedure.

Postoperatively, VCSS scores improved in over 80% of those undergoing TIPP. It must be remembered that patients chosen in this study to undergo TIPP have severe venous disease. In these patients, we assume that other treatment options have more drawbacks relative to TIPP.

Table 2 presents a summary of major series reported in the English-language literature. Most of these series report good results with TIPP that are non-inferior when compared to other surgical techniques. The avoidance of general or regional anesthesia and fewer incisions make TIPP an attractive surgical alternative. Different to most other studies describing TIPP, our unit performs this procedure in order to offer the best possible solution for a select group of patients. Pain is a significant drawback that should be taken into account when offering TIPP to patients who would otherwise benefit from alternative procedures.

**Table 2:** Selected Studies on Transilluminated Powered Phlebectomy (TIPP).

Study	Study Type	No. Patients	Comments
Spitz et al.^2^	Case series	36	Office procedure including 33 saphenous vein ablations; patients comfortable without severe pain
Cheshire et al.^3^	Uncontrolled study	114	All but 13 patients underwent combined TIPP with saphenous vein ablation or ligation. At 6 weeks: 1 patient dead from myocardial infarction; 1 patient with deep vein thrombosis. Other complications affecting limbs: nerve damage in 43; ecchymosis in 33; swelling in 20; hematoma in 14; pain in 5; cellulitis in 4
Scavée et al.^4^	Case series	40	More hematomas reported with TIPP compared to stab avulsion phlebectomy (57% versus 22%). No differences in pain at 7 days and 6 weeks
Aremu et al.^5^	Randomized controlled study	88	88 TIPP patients compared with 100 conventional stab avulsion surgery patients Mean number of incisions: 5 TIPP versus 29 conventional No differences in pain, bruising, cellulitis, or numbness over time At 6 weeks, no differences noted in nerve injury, residual veins, cosmetic score, and overall satisfaction
Chetter et al.^6^	Randomized study	29	29 TIPP patients compared to 33 multiple stab incision phlebectomy patients Low number of TIPP incisions, but accompanied by extensive bruising, increased pain, and reduced quality of life
Akesson^7^	Case series	21	Pain decreased to baseline within 2 weeks
Franz et al.^8^; Franz et al.^9^	Case series	339^8^/431^9^	Staged procedure for saphenous vein ablation patients Following TIPP, 99.7% reported good outcome and procedure satisfaction
Kim et al.^10^	Case series	299	447 Limbs (TIPP) Complications reported: cellulitis, 2.2%; hematoma, 3.5%; cutaneous nerve damage, 2.2%; seroma, 2.9%
Obi et al.^11^	Case series	657	TIPP with saphenous vein ablation in most patients; hematomas reported in 7.8%
Passman et al.^12^	Case series	169	Combined TIPP procedure with saphenous vein stripping or endovenous ablation; more hematomas with TIPP compared to stab avulsion phlebectomy

Certain limitations should be taken into account. All the patients included in this analysis underwent surgery performed by one surgeon. One may conclude that differences seen in pain intensity in this series compared to that reported by others may be surgeon-/technique- and anesthesia-dependent. This is true for pain experienced during surgery, but it does not explain why the degree of pain experienced by patients in the weeks afterwards should be different, as reported by Chetter et al.^6^ An alternative explanation for these differences may be the difference in patient population. Further studies of TIPP should concentrate on both short- and long-term pain. These limitations are probably true in other studies on TIPP reported in the literature. We believe that the TIPP procedure for our patients is the best procedure for them in the long run, despite the significant postoperative pain they experience.

In conclusion, TIPP procedures allow surgical treatment of extensive varicosities in patients who otherwise have no real alternative for treatment. However, as this study shows, many patients report significant pain during surgery and also postoperatively. Patients referred for TIPP procedures should be well informed about the limitations of this procedure.

## Abbreviations

TIPP: transilluminated powered phlebectomy
VCSS: Venous Clinical Severity Score.

## References

[1] Chen S, Zeng Q, Fu Q, Li F, Zhang M, Zhao Y (2019). Transilluminated powered phlebectomy in the treatment of large area venous leg ulcers: a case-control study with 3 years follow-up. Microcirculation.

[2] Spitz GA, Braxton JM, Bergan JJ (2000). Outpatient varicose vein surgery with transilluminated powered phlebectomy. Vasc Surg.

[3] Cheshire N, Elias SM, Keagy B (2002). Powered phlebectomy (TriVex) in treatment of varicose veins. Ann Vasc Surg.

[4] Scavée V, Lesceu O, Theys S, Jamart J, Louagie Y, Schoevaerdts JC (2003). Hook phlebectomy versus transilluminated powered phlebectomy for varicose vein surgery: early results. Eur J Vasc Endovasc Surg.

[5] Aremu M, Mahendran B, Butcher W (2004). Prospective randomized controlled trial: conventional versus powered phlebectomy. J Vasc Surg.

[6] Chetter IC, Mylankal KJ, Hughes H, Fitridge R (2006). Randomized clinical trial comparing multiple stab incision phlebectomy and transilluminated powered phlebectomy for varicose veins. Br J Surg.

[7] Akesson H (2008). Transilluminated powered phlebectomy: a clinical report. Phlebology.

[8] Franz RW, Knapp ED (2009). Transilluminated powered phlebectomy surgery for varicose veins: a review of 339 consecutive patients. Ann Vasc Surg.

[9] Franz RW, Hartman JF, Wright ML (2012). Treatment of varicose veins by transilluminated phlebectomy surgery: a 9-year experience. Int J Angiol.

[10] Kim JW, Han JW, Jung SY, Lim MS, Jung JP, Cho JW (2013). Outcome of transilluminated powered phlebectomy for varicose vein: review of 299 patients (477 limbs). Surg Today.

[11] Obi AT, Reames BN, Rook TJ, Michigan Vein Health Program (2016). Outcomes associated with ablation compared to combined ablation and transilluminated powered phlebectomy in the treatment of varicose veins. Phlebology.

[12] Passman MA, Dattilo JB, Guzman RJ, Naslund TC (2007). Combined endovenous ablation and transilluminated powered phlebectomy: is less invasive better?. Vasc Endovasc Surg.

[13] Spitz G (2011). Transilluminated powered phlebectomy in an office setting: procedural considerations and clinical outcome. J Endovasc Ther.

[14] Passman M (2007). Transilluminated powered phlebectomy in the treatment of varicose veins. Vascular.

[15] Arumagasamy M, McGreal G, O’Connor A, Kelly C, Bouchier-Hayes D, Leahy A (2002). The technique of transilluminated powered phlebectomy – a novel, minimally invasive system for varicose vein surgery. Eur J Vasc Endovasc Surg.

[16] Vasquez MA, Munschauer CE (2008). Venous Clinical Severity Score and quality of life assessment tools: application to vein practice. Phlebology.

[17] Gloviczki P, Comerota AJ, Dalsing MC (2011). The care of patients with varicose veins and associated chronic venous diseases: clinical practice guidelines of the Society for Vascular Surgery and the American Venous Forum. J Vasc Surg.

[18] Chou R, Gordon DB, de Leon-Casasola OA (2016). Management of postoperative pain: a clinical practice guideline from the American Pain Society, the American Society of Regional Anesthesia and Pain Medicine, and the American Society of Anesthesiologists’ Committee on Regional Anesthesia, Executive Committee, and Administrative Council. J Pain.

[19] Hudson BF, Ogden J, Whiteley MS (2015). Randomized controlled trial to compare the effect of simple distraction interventions on pain and anxiety experienced during conscious surgery. Eur J Pain.

